# Multimodal data fusion AI model uncovers tumor microenvironment immunotyping heterogeneity and enhanced risk stratification of breast cancer

**DOI:** 10.1002/mco2.70023

**Published:** 2024-12-11

**Authors:** Yunfang Yu, Gengyi Cai, Ruichong Lin, Zehua Wang, Yongjian Chen, Yujie Tan, Zifan He, Zhuo Sun, Wenhao Ouyang, Herui Yao, Kang Zhang

**Affiliations:** ^1^ Guangdong Provincial Key Laboratory of Malignant Tumor Epigenetics and Gene Regulation, Department of Medical Oncology Breast Tumor Centre, Phase I Clinical Trial Centre, Sun Yat‐sen Memorial Hospital, Sun Yat‐sen University Guangzhou China; ^2^ Faculty of Medicine Macau University of Science and Technology Taipa Macao China; ^3^ Faculty of Innovation Engineering Macau University of Science and Technology Taipa Macau China; ^4^ Dermatology and Venereology Division, Department of Medicine Solna, Center for Molecular Medicine Karolinska Institute Stockholm Sweden; ^5^ Institute for Advanced Study on Eye Health and Diseases Wenzhou Medical University Wenzhou China; ^6^ Guangzhou National Laboratory Guangzhou China; ^7^ Zhuhai International Eve Center Zhuhai People's Hospital and the First Affiliated Hospital of Faculty of Medicine, Macau University of Science and Technology and University Hospital Zhuhai China

**Keywords:** artificial intelligence, breast cancer, immune–metabolic subtypes, prognostic prediction, tumor microenvironment

## Abstract

Breast cancer is the leading cancer among women, with a significant number experiencing recurrence and metastasis, thereby reducing survival rates. This study focuses on the role of long noncoding RNAs (lncRNAs) in breast cancer immunotherapy response. We conducted an analysis involving 1027 patients from Sun Yat‐sen Memorial Hospital, Sun Yat‐sen University, and The Cancer Genome Atlas, utilizing RNA sequencing and pathology whole‐slide images. We employed unsupervised clustering to identify distinct lncRNA expression patterns and developed an AI‐based pathology model using convolutional neural networks to predict immune–metabolic subtypes. Additionally, we created a multimodal model integrating lncRNA data, immune‐cell scores, clinical information, and pathology images for prognostic prediction. Our findings revealed four unique immune–metabolic subtypes, and the AI model demonstrated high predictive accuracy, highlighting the significant impact of lncRNAs on antitumor immunity and metabolic states within the tumor microenvironment. The AI‐based pathology model, DeepClinMed‐IM, exhibited high accuracy in predicting these subtypes. Additionally, the multimodal model, DeepClinMed‐PGM, integrating pathology images, lncRNA data, immune‐cell scores, and clinical information, showed superior prognostic performance. In conclusion, these AI models provide a robust foundation for precise prognostication and the identification of potential candidates for immunotherapy, advancing breast cancer research and treatment strategies.

## Graphical abstract

This study introduces an advanced cohort research on breast cancer prognosis, revealing four distinct immune–metabolic subtypes with notable survival differences. Using cutting‐edge AI techniques, we developed a pathology‐based model integrating a modified ResNet50 network with an attention‐gated mechanism for subtype prediction. Additionally, our multimodal AI model, incorporating deep learning and data fusion, accurately predicts prognostics scores.

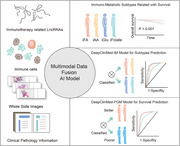

## 1 INTRODUCTION

Breast cancer is the most prevalent cancer among women globally. It poses a significant challenge given that 30–40% of early‐stage patients experience recurrence and metastasis, resulting in an advanced‐stage 5‐year survival rate dropping below 23%.^1^, ^2^, ^3^ Although immunotherapy shows promise, its clinical benefits are limited to a subset of patients, which underscores the heterogeneity in breast cancer therapy and highlights the need to identify prognostic factors for effective immune checkpoint inhibitor treatment.^4^, ^5^

Long noncoding RNAs (lncRNAs) have garnered considerable attention for their multifaceted roles across diverse biological processes, such as proliferation, metabolism, drug resistance, and maintenance of stemness.^6^, ^7^, ^8^ Notably, previous research has underscored the significant effect of lncRNAs on antitumor T cell immunity and specific immunotherapy subtypes, highlighting their potential as predictive biomarkers for therapy response and overall survival (OS).^9^, ^10^, ^11^ The comprehensive understanding of lncRNA involvement in breast cancer assumes critical importance, not only for elucidating the intricacies of the tumor microenvironment (TME) but also for the development of precise therapeutic strategies tailored to this complex disease.

Furthermore, the intricate interplay between lncRNA, immune cells, and metabolic dynamics within the TME emerges as a pivotal determinant in cancer biology. This interrelation, coupled with the recognized metabolic reprogramming inherent to cancer, profoundly influences the TME, significantly sculpting the landscape of antitumor immunity.^12^ Within this intricate milieu, infiltrating immune cells and tumor‐associated stromal cells surface as pivotal orchestrators, with considerable influence over tumor progression, treatment responses, and effectiveness of immunotherapy. Recent investigations increasingly underscore the pivotal role of metabolic states within the TME in modulating antitumor immunity.^13^, ^14^ However, while substantial attention is directed toward unveiling insights into tumor biology and prognostic implications, the predominant focus remains entrenched in mechanistic understanding, relegating clinical applications to a relatively unexplored domain within the existing literature.^15^, ^16^ Bridging this translational gap necessitates a concerted effort to convert metabolic insights into clinically applicable methodologies, thereby fostering transformative strides in cancer therapeutics and patient care.

Despite extensive research on the tumor immune microenvironment's link to tumor subtypes and prognosis, there is still a notable gap in accessible quantification methods that use small tissue samples for comprehensive TME assessment. Pathology‐based artificial intelligence (AI), which relies solely on pathology slides, offers a novel tool for prognosis prediction, and according to recent studies, it exhibits robust efficacy in prognostication,^17^ biomarker prediction,^18^ and treatment decisions.^19^ However, prevailing approaches often focus on singular data modalities, neglecting the potential gains from integrating diverse data sources. Integrating these modalities presents opportunities for heightened accuracy and the discovery of novel patterns, pivotal for explaining patient outcome variations or treatment resistance.^20^ Some strides have been made in AI's integration of multimodal data, showcasing superior precision in treatment decisions, particularly in combining genomic and image data.^21^, ^22^, ^23^, ^24^ AI‐driven analyses quantifying posttreatment changes demonstrate potential applicability across solid tumors, promising advancements in leveraging various image data types, including pathology images, to enhance TME quantification.

In this study, we aimed to conduct an analysis of immunotherapy‐associated lncRNAs and immune cells in breast cancer, employing unsupervised clustering for classification. Furthermore, we developed an AI‐based model utilizing pathology data to predict the immune–metabolic subtypes of breast cancer. The delineation of immune subtypes in breast cancer linked to metabolic signatures holds promise for identifying potential candidates for immunotherapy. Additionally, we implemented a multimodal model that demonstrated state‐of‐the‐art performance in prognostic prediction in patients with breast cancer. These findings have the potential to significantly contribute to the progress of breast cancer immunotherapy.

## 2 RESULTS

### 2.1 Integrative analysis of lncRNA expression and immune cell composition in breast cancer immunotherapy response

**FIGURE 1:**
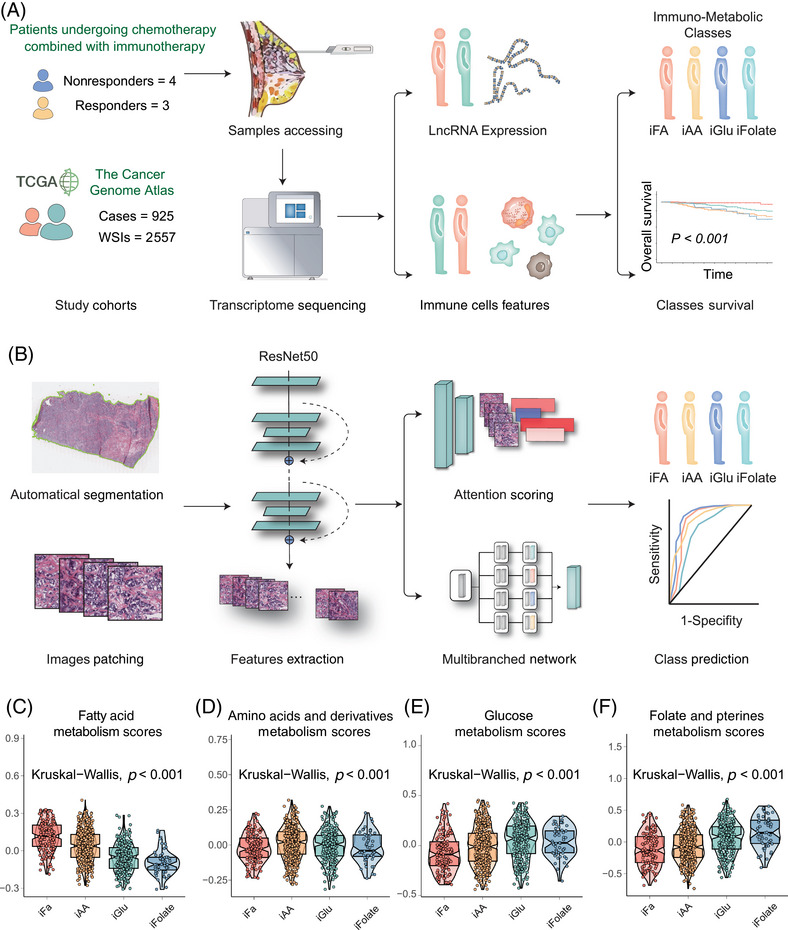
Immune–metabolic subtypes in breast cancer and AI‐based pathology‐driven model for subtypes classification. (A) Graphical summary of the discovery of immune–metabolic subtypes. Samples were obtained from seven patients undergoing chemotherapy combined with immunotherapy (four nonresponders and three responders) in the SYSMH cohort and subjected to transcriptome sequencing. Analysis of lncRNA expression signatures and immune cell characteristics led to the identification of immune–metabolic subtypes with significant survival differences. (B) Construction of the AI‐based pathology model for predicting immune–metabolic subtypes. Digitized high‐resolution histology slides were automatically segmented and converted into patches. Feature extraction using ResNet50 was followed by scoring through a gated attention mechanism. Patients were classified into immune–metabolic subtypes, and the model's performance was evaluated. (C–F) Violin plots illustrating the distribution of major metabolism scores across each immune–metabolic subtype (Kruskal–Wallis test, *p* < 0.001).

In this investigation, we included seven breast cancer patients undergoing immunotherapy at Sun Yat‐sen Memorial Hospital of Sun Yat‐sen University (SYSMH). Employing transcriptome sequencing, we analyzed lncRNA expression profiles in patients with distinct treatment responses to chemotherapy combined with immunotherapy (three responders vs. four nonresponders). A meticulous screening identified 198 lncRNAs associated with diverse treatment responses (Figure S1A,B). Subsequently, we applied unsupervised clustering using deep learning to the breast cancer samples of 925 patients in The Cancer Genome Atlas Program (TCGA) with sequenced lncRNAs, establishing a lncRNA‐based breast cancer immunophenotyping system (Figures 1A,B and S1C). The clustering revealed three distinct lncRNA‐based clusters, namely, lncRNA‐cluster 1 (369 patients), lncRNA‐cluster 2 (334 patients), and lncRNA‐cluster 3 (222 patients).

Building on previous findings^9^ associating lncRNA and CD8^+^ T cells with breast cancer classification and response to immunotherapy, we extended investigation to explore correlations between lncRNA and other components of the TME. Employing single sample gene set enrichment analysis (ssGSEA) analysis, we identified 28 immune cell types in the TME in the 925 patients. Unsupervised clustering on the TCGA cohort revealed predominant separation into two immune‐cell clusters (Figure S1D).

### 2.2 Deciphering tumor metabolism via lncRNA‐immune cell interplay

A previous study demonstrated the prognostic value between T‐cell infiltration and lncRNA signature.^9^ The individuals showing both an active immune response and significant immune‐cell infiltration had higher expression levels of immune molecules than those with a functional immune response but lower levels of immune‐cell infiltration; this delineation identified individuals within the “immune‐active” and “immune‐exclusion” tumor groups, respectively. Furthermore, the patients exhibiting low expression of immunotherapy‐associated lncRNAs were stratified based on their immune‐cell infiltration levels into the “immune‐dysfunctional” and “immune‐desert” groups.

Utilizing the aforementioned lncRNA profiles in conjunction with immune status, we developed a two‐dimensional index classifying the patients into four subtypes as an extension of our former studies, which demonstrated distinct metabolic signatures. Hence, the amalgamation of “lncRNA‐cluster 1 and immune cell cluster 1” characterized the “immune‐active class” subtype. Either “lncRNA‐cluster 2 and immune cell cluster 1” or “lncRNA‐cluster 1 and immune cell cluster 2” represented the “immune‐exclusion class” subtype. Next, “lncRNA‐cluster 2 and immune cell cluster 2” or “lncRNA‐cluster 3 and immune cell cluster 1” identified the “immune‐dysfunctional” subtype. Finally, “lncRNA‐cluster 3 and immune cell cluster 2” delineated the “immune‐desert” subtype.

To uncover the underlying mediation between lncRNA–immune subtypes and immune status, we conducted gene set enrichment analysis (GSEA) pathway analyses across the four subtypes (Kruskal–Wallis test, *p* < 0.001; Figure 1C–F). Intriguingly, distinct metabolic statuses differentiated these subtypes. The “immuno–fatty acid (iFA)” subtype, denoting activation of FA metabolism, was attributed to the “immune‐active class” subtype. In contrast, the “immuno–amino acid (iAA)” subtype associated with the “immune‐exclusion class” displayed enrichment in AA metabolism. The “immuno–glucose (iGlu)” subtype, indicative of the “immune‐dysfunctional” tumors, exhibited enriched Glu metabolism. Last, the “immune‐desert” subtype revealed upregulated folate and pterin metabolism, which was identified as “immuno–folate (iFolate)” subtype.

**FIGURE 2:**
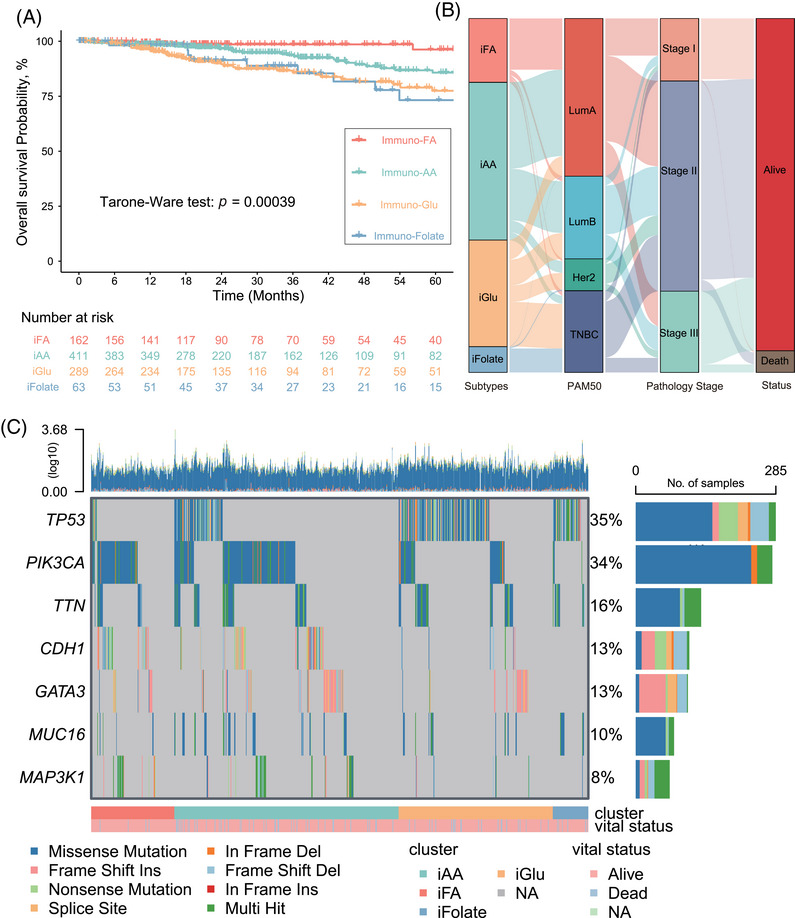
Construction of the four immune–metabolic subtypes in breast cancer. (A) Kaplan–Meier survival analysis of 925 patients, comparing disease‐free survival among lncRNA‐metabolism subtypes (log‐rank test, *p* < 0.001). (B) Sankey plot depicting the relationship between lncRNA‐metabolism subtypes, PAM50 subtype, pathology stage, and patient survival status. (C) Oncoplot showcasing somatic mutation profiles for each immune–metabolic subtype. iAA, immuno–amino acid subtype; iFA, immuno–fatty acid subtype; iFolate, immuno–folate subtype; iGlu, immuno–glucose subtype.

Crucially, OS concerning immunotherapy showcased a significant variance across these subtypes. The immuno–FA subtype manifested the most substantial OS benefit from immunotherapy (Tarone‐Ware test, *p* = 0.00039; Figure 2A), implying a potential link between Fatty acid metabolism and patient prognosis. The immuno–Glu subtype had the shortest OS, indicating a potential correlation between folate and pterin metabolism and poorer prognosis. Sankey plotting revealed a predominant association of the immuno‐FA and immuno‐AA tumor subtypes with Lumina A and Lumina B subtypes according to PAM50 subtypes. Conversely, the immuno‐Glu and immuno‐Folate subtypes predominantly correlated with TNBC, aligning with later‐stage tumors and poorer prognostic outcomes (Figure 2B).

Furthermore, we depicted somatic mutation landscapes across the four subtypes through OncoPrint analysis. Notably, PIK3CA mutations were predominant in the immuno–FA (54%; Figures S3A and S5A) and immuno–AA (41%; Figures S3B and S5B) subtypes, while TP53 mutations were more prevalent in the other two subtypes, especially in the immuno–folate subtype (82%; Figures S3C,D and S4). This finding suggests divergent genetic heterogeneity driving these subtypes and potentially contributing to the heterogeneity in the TME and metabolic states.

### 2.3 Distinct metabolic profiles, immune landscapes, and functional traits in tumor subsets

**FIGURE 3:**
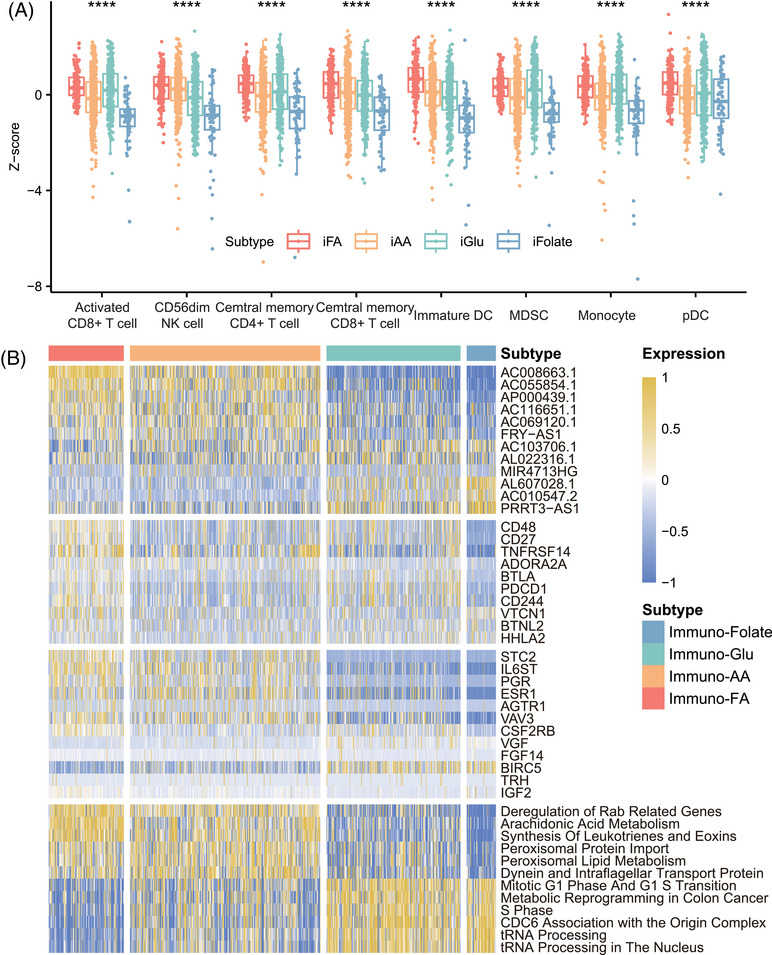
Distinct metabolic profiles, immune landscapes, and functional traits of immune–metabolic subtypes in breast cancer. (A) Boxplots showing distinct patterns of immune cell infiltration for each subtype, with jitter points representing individual patient expression levels. (B) Heatmaps of the top three signatures for each subtype, including immunotherapy‐associated lncRNA expression, immune checkpoint gene expression, immune‐related gene expression, and enriched pathways from GSEA analysis. Each cell reflects the expression level or score for each patient.

We further analyzed the distribution of immune cells in these four subtypes. We found that high infiltration of some cells (activated CD8+ T cells, natural killer [NK] cells, central memory CD4^+^ T cells, central memory CD8^+^ T cells) was involved in immuno–FA subtype, highlighting the correlation between immune‐active state and FA metabolism state (Figures 3A and S6). However, we found that in immuno–Glu subtype that also identified as immune‐dysfunctional group, high infiltration of some cells (myeloid‐derived suppressor cells [MDSCs], T follicular helper cells, type 1 T helper cells, macrophages) was also involved, suggesting that dysfunction of the glucose metabolism may be involved in the immune‐dysfunctional state (Figure S6). These results confirmed the correlation underlying the four metabolism subtypes and immune status. However, some of the antitumor effective immune cells were both high in immuno–FA and immuno–Glu subtype, while patients in immuno–Glu subtype showed poorer prognosis than immuno–FA and immuno–AA, even when fewer immune cells demonstrated high level in immuno–AA. This phenomenon also indicated that level of immune cells infiltration could not show credible immune state and demonstrated that subtypes with higher dimension is necessary.

Next, we investigated the relationship between immune checkpoint sets and immune genes in the four metabolism subtypes. The samples in immuno–FA subtype showed higher immune checkpoints gene expression than the samples in immuno–AA subtype, suggesting a benefit of immunotherapy (Figure S7). The samples in immuno–FA subtype were associated with high expression levels of CD48, CD27, and TNFRSF14. The samples in immuno–Glu subtype were associated with high BLTA, PDCCD1, and CD244 expression (Figure 3B). As for the expression of other immune‐related genes, the patients in immuno–FA subtype also had a higher STC2, PGR, and IL6ST expression (Figure 3B). Moreover, the samples in immuno–folate subtype had worse prognosis and lower immune gene expression than those in the other three clusters (Figure S7).

Furthermore, we employed GSEA to extend our exploration beyond the primary metabolic pathways, examining additional pathways associated with the distinctive metabolic states identified across the four subtypes. Within immuno–FA subtype, a predominant association was observed with lipid metabolism pathways, such as arachidonic acid metabolism (Figure 3B). Noteworthy within this subtype was the notable appearance of immune‐related pathways, notably the IL‐6 signaling pathway and complement pathway, hinting at an intricate interplay between metabolism and immune modulation (Figure S7). Conversely, immuno–folate subtype exhibited a distinct enrichment profile primarily linked to pathways critical in tRNA processing and transcriptional processes (Figure 3B). This divergence underscores the diverse biological foundations characterizing these metabolic subtypes, suggesting varying mechanisms governing tumor progression and immune response modulation within these distinct subtypes.

### 2.4 AI‐based pathology model for precise prediction of immune–metabolism subtypes

**FIGURE 4:**
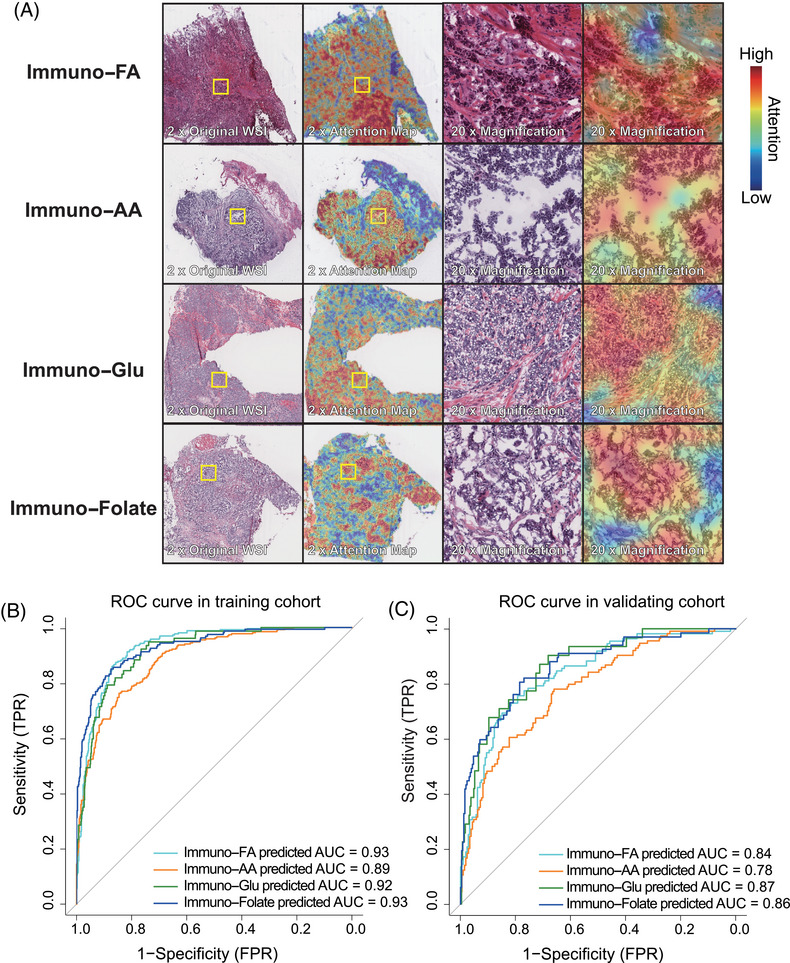
Development of an AI‐based pathology‐driven prediction model for immune–metabolic subtypes in breast cancer. (A) Pathology slide examples for each subtype with attention heat maps. Each case displays an image of the original patches and another with the model's attention heat map overlaying the original H&E WSI. The overlay ranges from crimson (high attention, high diagnostic relevance) to navy (low attention, low diagnostic relevance). The right panel shows a higher magnification of the left panel. (B) ROC curves demonstrating the performance of the AI‐based pathology‐driven prediction model in the training cohort. (C) ROC curves demonstrating the performance of the AI‐based pathology‐driven prediction model in the validation cohort. AUC, area under the curve; FPR, false‐positive rate; TPR, true‐positive rate.

In light of the intricacies in delineating the immune–metabolic subtypes, we developed a pathology‐based AI model, DeepClinMed‐IM (deep learning–based clinical immune–metabolic subtypes), for subtype prediction (Figure 1B). Thorough exploration of the hyperparameter space for the transfer learning model, incorporating a linear mapping to connect attention at its penultimate layer using ResNet50, led to the identification of the most optimal configuration. This resulted in accurate predictive performance for categorizing the four subtypes within both the training and the validation cohorts. After performing fivefold cross‐validation, the model with best performance for each cohort was identified and presented. For each slide, the model's attention can be visualized, showing captured regions of cells for subtype discrimination. A specific image with summarized regions of interest was demonstrated as Figure 4A.

A thorough stratified analysis was conducted to evaluate the prediction model's performance within each subtype, and yielded consistent and robust results. The receiver operating characteristic (ROC) curves depicted impressive discrimination, showcasing the area under the curve (AUC) values of 0.93 (immuno–FA subtype), 0.89 (immuno–AA subtype), 0.92 (immuno–Glu subtype), and 0.93 (immuno–folate subtype) within the training cohort (Figure 4B). The AUC values remained notably high within the validation cohort, registering at 0.84 for immuno–FA subtype, 0.78 for immuno–AA subtype, 0.87 for immuno–Glu subtype, and 0.86 for immuno–folate subtype (Figure 4C). The successful deployment of this pathology‐based AI model underscores its efficacy in accurately predicting immune–metabolism subtypes, demonstrating promising performance and generalizability across distinct cohorts.

### 2.5 AI‐based multimodal model for precise prediction of prognostics

**FIGURE 5:**
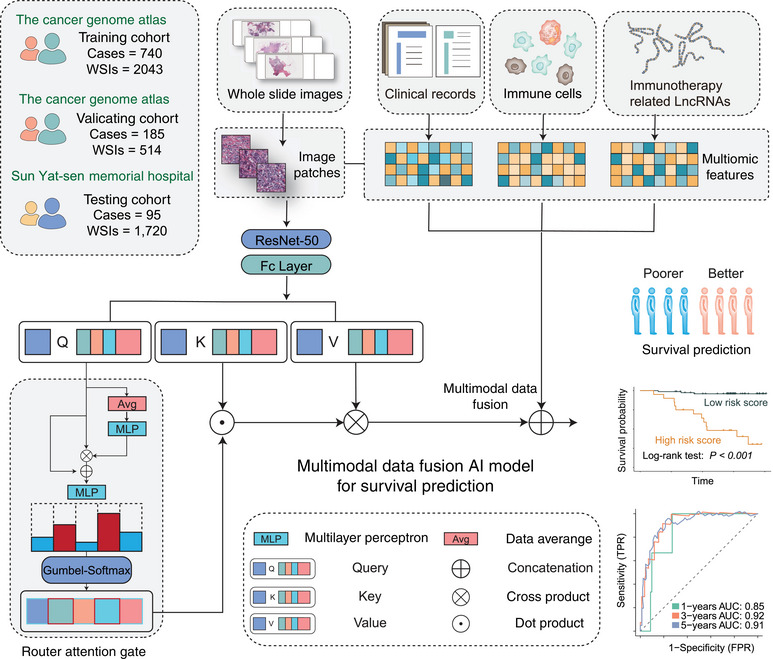
Development of an AI‐based multimodal prediction model for disease‐free survival. (A) Construction of the AI‐based multimodal model using data from the SYSMH and TCGA cohorts, which include whole‐slide images (WSIs), multiomic features, and clinical records. WSIs were processed through ResNet‐50 and a fully connected layer to extract features. These features were then combined with multiomic and clinical data using attention mechanisms and a multilayer perceptron (MLP). The model predicts patient survival across training, validation, and testing cohorts, with performance evaluated via ROC curves.

Expanding on the previously mentioned model, we innovatively integrated multimodal inputs, including pathology images, lncRNA data, immune‐cell ssGSEA scores, and clinical information seamlessly. This novel addition of a QR played a pivotal role in refining the fusion approach, contributing to a more robust prognostics prediction model named DeepClinMed‐PGM (deep learning–based multimodal clinical pathology genomics) (Figure 5). The integration of diverse data types through the enhanced fusion paradigm significantly bolstered the predictive capabilities across all cohorts, showcasing substantial advancements in disease prognosis. Likewise, datasets were randomly split into five equal parts according to the label distribution, and a fivefold Monte Carlo cross‐validation was conducted.

**FIGURE 6:**
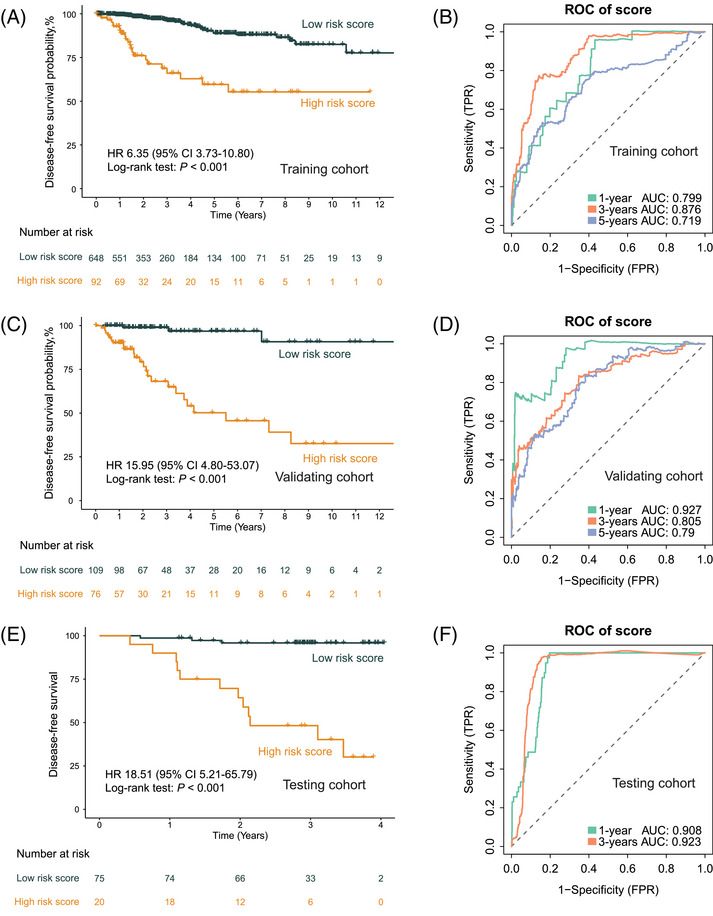
Evaluation of AI‐based multimodal prediction model for disease‐free survival. (A, C, and E) Kaplan–Meier curves comparing predicted high‐ and low‐risk patients in the training cohort (*n* = 740), internal validation cohort (*n* = 185), and independent testing cohort (*n* = 95). (B, D, and F) ROC curves for survival prediction by the AI‐based multimodal model in the training cohort (*n* = 740), internal validation cohort (*n* = 185), and independent testing cohort (*n* = 95). AUC, area under the curve; FPR, false‐positive rate; HR, hazard ratio; TPR, true‐positive rate.

The resultant model demonstrated exceptional performance metrics, exhibiting a concordance index of 0.82 in the training cohort (*n* = 740, sourced from TCGA), 0.83 in the validating cohort (*n* = 185, also from TCGA), and an impressive 0.90 in the independent testing cohort (*n* = 95, the SYSMH cohort), and Table S1 shows the clinicopathologic characteristics of the patients in the testing cohort. The survival analysis showcased the model's robust performance in stratifying distinct disease‐free survival (DFS) risk profiles across the various cohorts, with hazard ratios (HRs) values of 6.35 and 15.95 observed in the training (*n* = 740, 95% CI 3.73–10.80, Log‐rank test: *p <* 0.001; Figure 6A) and validation (*n* = 185, 95% CI 4.80–53.07, Log‐rank test: *p <* 0.001; Figure 6C) datasets from TCGA, respectively. In the independent testing cohort sourced from the SYSMH cohort, the HR value was 18.51 (*n* = 95, 95% CI 5.21–65.79, Log‐rank test: *p <* 0.001; Figure 6E).

Notably, the AUC values at 1, 3, and 5 years DFS were noteworthy indicators of predictive accuracy. Within the training cohort (*n *= 740; Figure 6B), the AUC values at 1, 3, and 5 years stood at 0.80, 0.88, and 0.72, respectively, demonstrating the model's robustness over various time frames. Similarly, in the validation cohort (*n* = 185; Figure 6D), the AUC values at 1, 3, and 5 years were 0.93, 0.81, and 0.79, respectively, confirming the model's consistent predictive power. Importantly, the independent testing cohort (*n* = 95; Figure 6F) demonstrated compelling AUC values at 1 and 3 years, reaching 0.91 and 0.92 for DFS prediction, respectively.

Next, we compared the ROC curves of single‐omics and pathology slide‐based models with our DeepClinMed‐PGM model for prognostic prediction. While the DeepClinMed‐PGM model achieved a concordance index of 0.82–0.90, the single‐omics model only showed indices of 0.69 in the training cohort, 0.70 in the validation cohort, and 0.60 in the testing cohort. And in the training cohort (*n* = 740; Figure S8A), the AUC values at 1, 3, and 5 years were 0.80, 0.88, and 0.72, respectively. The validation cohort (*n* = 185; Figure S8B) had AUC values of 0.93, 0.81, and 0.79 at 1, 3, and 5 years. The testing cohort (*n* = 95; Figure S8C) showed AUC values of 0.91 and 0.92 at 1 and 3 years for DFS prediction. These results confirmed that our multimodal data based‐model DeepClinMed‐PGM showed consistently superior predictive power compared with the single‐omics model across all cohorts.

We also analyzed the weights assigned to each feature in our model. Clinical data like clinical stage and T stage showed high positive weights, indicating significant contributions, while age and PAM50 subtype had high negative weights, suggesting an inverse relationship to the model's output (Figure S9). Additionally, immune cells like Type 1 T helper cells and effector memory CD4 T cells had high positive weights, whereas eosinophils and Type 2 T helper cells had high negative weights (Figure S9). This detailed weight distribution provides insights into the roles of various features within the multimodal data‐based model framework, highlighting the model's ability to leverage the heterogeneity of multimodal omics for accurate predictions.

## 3 DISCUSSION

Our comprehensive analysis of lncRNA and immune‐cell profiling in breast cancer has led to the establishment of a novel classification system that integrates genomic, metabolic, and immunological dimensions. The study identified four distinct immune–metabolic subtypes based on metabolic status, namely, immuno–FA, immuno–AA, immuno–Glu, and immuno–folate, shedding new light on the molecular heterogeneity of breast cancer. Notably, the AI‐based pathology model utilizing convolutional neural network (CNN) technology, DeepClinMed‐IM, exhibited exceptional predictive performance in both the training and the validation cohorts. Furthermore, another model developed, DeepClinMed‐PGM, leveraging multimodal data encompassing clinical information, transcriptome, and pathology images, achieved noteworthy accuracy in predicting prognostics. This integrated approach not only enhances the understanding of breast cancer subtypes but also provides a robust foundation for precise prognostication and potential immunotherapeutic candidate identification, marking a significant advancement in breast cancer research and treatment strategies.

LncRNAs play a pivotal role in orchestrating breast cancer progression and shaping the immune microenvironment.^7^, ^8^ During the initiation of breast cancer, evasive mechanisms allow cancer cells to circumvent immune surveillance. As tumors progress, bidirectional communication between tumor cells and the TME, either contact dependent or independent, influences the production of cytokines, contributing to immunosuppression and polarization of antitumor immune responses within the microenvironment. lncRNAs intricately regulate various signals governing immune and cancer cell crosstalk, thereby influencing processes such as tumorigenesis,^25^, ^26^ tumor invasion,^27^ and epithelial–mesenchymal transition^26^ by participating in various biological processes such as neutrophil recruitment, macrophage polarization, NK cytotoxicity, and T‐cell function. This research established a novel correlation among lncRNA expression, immune infiltration, and metabolism. Previous studies have demonstrated the feasibility of classifying tumor patients based on lncRNA signatures and T‐cell infiltration.^9^ This study not only broadened our inquiry by integrating information from 28 immune‐cell types, but also unveiled a promising link among metabolism status, lncRNA expression, and immune infiltration in patients following immunotherapy.

Metabolic reprogramming, recognized as a hallmark of cancer, is closely linked to immune evasion, tumor resistance, recurrence, and progression. Disturbances in metabolic status, such as aerobic glycolysis in tumor cells,^28^, ^29^ have been observed in various solid tumors and are associated with poor prognosis.^30^, ^31^ Additionally, metabolic mechanisms, including glutamine, glutathione, and serine metabolism, influence both the metabolic features of the TME and immune‐cell functions.^29^, ^32^, ^33^ Although previous studies have highlighted the importance of TME metabolic features and their impact on immunotherapy, few have focused on the practical clinical application of these metabolic characteristics. This study identified novel tumor subtypes associated with immunotherapy benefits, revealing a correlation between the metabolic preferences of breast cancer and patient prognosis. It presented a comprehensive view of the metabolism status and antitumor immunity in breast cancer cohort, categorized by lncRNA subtypes, demonstrating the potential for predictive applications in antitumor strategies.

This study also validated the integration of AI algorithms to explore the complex correlations among pathology, lncRNA, and the TME in breast cancer. This represents a significant advance in leveraging image‐based AI and marks an initial breakthrough in employing deep learning methodologies to quantify pathological images, lncRNA profiles, and immune‐cell landscapes within breast cancer immunophenotyping. Our previous investigations have notably highlighted specific imaging features in breast cancer, particularly radiomic images,^34^, ^35^ demonstrating potential for noninvasive lncRNA quantification using radiomics. Additionally, AI‐driven models have shown promise in predicting origins of unknown primary cancers and aiding pathologic assessments during immunotherapy clinical trials using pathology images.^36^ Despite some studies showcasing the feasibility of pathology‐based models in identifying transcriptional features and making predictions,^37^, ^38^ there has been limited exploration of their potential for quantifying lncRNA using pathology images and their clinical utility in immunotherapy setting. This study bridged this gap by establishing a crucial connection between breast cancer immune subtypes, pathological image traits, and the exploratory quantification of lncRNA and immune‐cell distributions. This pioneering effort achieved quantification of lncRNA and immune cell–based breast cancer immunophenotyping alongside spatial distribution within pathological images, enabling early, efficient, and accurate prognostic predictions for breast cancer patients undergoing immunotherapy.

Moreover, we strove to utilize a multimodal approach to constructing an AI model with superior predictive performance. In contrast to traditional single‐omics methods, multimodal approach offers unique advantages in advancing precision medicine, deepening our understanding of disease biology, and enhancing therapeutic efficacy. By integrating genomics, transcriptomics, pathology, and radiomics, these comprehensive datasets provide a holistic understanding, facilitating exploration of disease progression and treatment response.^39^, ^40^ One strength of multimodal approaches lies in identifying biomarkers across different layers, improving prediction accuracy.^41^ Considering diverse biological processes enhances the ability to capture disease features. The use of multimodal approaches may not be ideal for diseases characterized by significant heterogeneity; however, these approaches are effective in addressing changes at multiple levels and facilitating personalized medicine. The integration of information from various sources, including genotype, phenotype, pathology, and metabolomics, enhances our understanding of a patient's disease characteristics. In this study, the integration of pathology images and transcriptomic data, specifically focusing on lncRNA and immune‐cell features, enabled precise prediction of breast cancer prognosis. This integrated approach not only enhanced prognosis accuracy but also contributed to unraveling molecular and immune interactions in breast cancer, which would assist clinical decisions like PD1/PD‐L1 by evaluating immune status of the patients. Future efforts could also consider prospective studies or the inclusion of data from other tumor types for wider application range.

In this study, several limitations need consideration. First, the retrospective cohort design inherently impedes the establishment of causal relationships, given potential biases introduced by historical data. Concerns also arise about RNA degradation in pathology slides and the relatively short follow‐up time, which may impact the reliability of the findings. And therefore, the cohort from our hospital was not set as additional evaluation cohort as the pathology images‐based model. Second, incorporating populations from both TCGA and our hospital introduced inherent heterogeneity, encompassing differences in ethnicity and age distribution. Recognizing and addressing these diversities is crucial for accurately interpreting study outcomes. Third, as a multimodal study, only patients with both sequencing data and pathology slides were enrolled, resulted that the size of cohort from our hospital was not large enough. A larger scale of validation in future is necessary to enhance the credibility of the model. Furthermore, the conclusion of the study underscores the need for caution in inferring clinical utility. For example, our result showed that besides patients in immuno–Glu subtypes scored high in glucose metabolism, their also shared similar scores of acid metabolism states with immuno–AA. Therefore, these conclusions about underlying mechanism necessitate validation through prospective experiments, highlighting the importance of future studies with robust designs, larger and diverse patient cohorts, and extended follow‐up periods to enhance reliability and generalizability.

In conclusion, leveraging the predictive potential of lncRNAs and immune cells in breast cancer treatment, this study identified immunotherapy‐related four immune–metabolic subtypes in breast cancer, revealing the interplay between lncRNAs, immune cells, and metabolism. Moreover, an AI‐driven model leveraging pathology data accurately predicted the immune–metabolic subtypes of breast cancer. Identifying immune subtypes linked to metabolic signatures holds promise for identifying potential candidates for immunotherapy. Additionally, our implemented multimodal model demonstrated exceptional performance in prognostic prediction for breast cancer patients. These findings hold significant potential for advancing breast cancer immunotherapy and clinical decision‐making, with substantial implications for enhancing clinical treatment strategies.

## 4 MATERIALS AND METHODS

### 4.1 Study design and patients

In this study, we conducted an individual patient‐level analysis involving 1,027 patients, utilizing validated whole‐slide image (WSI) and RNA‐sequencing data in accordance with the Transparent Reporting of a Multivariable Prediction Model for Individual Prognosis or Diagnosis guideline.^42^ Patients in this study were enrolled from SYSMH, Sun Yat‐sen University, and TCGA. The SYSMH cohort comprised 95 breast cancer patients who received chemotherapy and standard treatment between September 2019 and February 2022. Additionally, seven patients who received chemotherapy combined with immunotherapy in SYSMH were enrolled in this study for subtypes construction. And the TCGA cohort comprised 925 patients with breast cancer.

The study proceeded through several phases, initially involving the categorization of all patients into three cohorts. In the construction of immune–metabolic subtypes, RNA‐sequencing data from seven patients in the SYSMH cohort who received chemotherapy combined with immunotherapy were accessed and analyzed for subtypes signatures. And RNA‐sequencing data from patients with 925 breast cancer in the TCGA cohorts were used for exploring biological characteristics of the subtypes.

As the basis for building both of the AI models in this study, a training cohort and an internal validation cohort were defined, comprising 741 and 184 patients, respectively, diagnosed through TCGA via the Genomic Data Commons (GDC) Data Portal.^43^ And 95 patients in the SYSMH cohort was defined as an external testing cohort for both of AI models in this study. RNA‐sequencing and WSI data from both cohorts were obtained for further analysis. Clinical data from all the patients in both cohorts were accessed, including age, PAM50 subtype, classification of T (tumor), N (nodes), and clinical stage. Based on label distribution, the dataset was randomly divided into five equal parts, and a fivefold Monte Carlo cross‐validation was performed.

The inclusion criteria were as follows: (a) female sex, age at least 18 years, and histologically confirmed stage I to III invasive breast cancer; (b) treatment with surgery and pathologically confirmed breast cancer treatment; and (c) availability of WSI and RNA‐sequencing data pertaining to breast tumors. The exclusion criteria were as follows: (a) samples exhibiting poor‐quality or inadequate pathological results; (b) incomplete data on WSI and RNA‐sequencing features or follow‐up information; and (c) presence of previous or simultaneous other tumors.

The primary outcome assessed in this study were OS and DFS. OS was defined as the duration from the date of breast cancer diagnosis to the time of death from any cause, or the date of the last follow‐up visit, whichever came first. DFS was defined as the duration from the breast cancer surgery to the occurrence of the first relapse at any site, confirmation of metastatic disease, death from any cause other than breast cancer, or the date of the last follow‐up visit, whichever came first. The T and N stages were assessed through imaging techniques (magnetic resonance imaging, ultrasonography, or positron emission tomography) or clinical examination, while follow‐up procedures adhered to the recommendations outlined in the National Comprehensive Cancer Network and American Joint Committee on Cancer Staging Manual guideline.^44^

*Ethical considerations and approval*: This study adhered to the principles set forth in the Declaration of Helsinki and received approval from the Ethics Committee of SYSMH, Sun Yat‐sen University (Approval Number: SYSKY‐2024‐363‐01). As the study was retrospective in nature and utilized publicly available datasets, the necessity for informed consent from participants was waived by the Ethics Committee.

### 4.2 Procedures of RNA sequencing

The RNA‐sequencing data of 925 patients were acquired from the TCGA database, adhering to the standardized RNA‐sequencing analysis procedures outlined in the corresponding guidelines. Additionally, we retrospectively acquired samples from 95 patients at SYSMH and conducted transcriptome RNA‐sequencing analysis. RNA was isolated from all tumor tissue samples using the formalin‐fixed paraffin‐embedded (FFPE) RNeasy kit, followed by the extraction process. Subsequently, RNA quantification and assessment of RNA integrity were conducted to ensure the quality of the extracted RNA. Following RNA isolation, the RNA library preparation included RNA fragmentation, reverse transcription, and addition of adapters for sequencing. The resulting libraries were subjected to amplification and then subjected to high‐throughput sequencing. The raw sequencing data were generated thorough preprocessing steps, including quality control to remove low‐quality reads and eliminate adapter sequences. After alignment of the cleaned reads to a reference genome, transcript expression levels were quantified through read counting.

Total RNA was extracted from FFPE samples using the QIAGEN FFPE RNeasy kit (QIAGEN GmbH, Hilden, Germany). Tissue sections suspended in QIAzol were vortexed for well mixing. Then, chloroform was added to each sample, vortexed, and transferred into new tubes. The mixing samples were then spun at 12,000×*g* for 15 min at 4°C, and the upper clear phase containing RNA was transferred. Subsequent extraction was performed using the Qiagen QIAsymphony in accordance with the recommended protocol. Subsequently, RNA quantification was performed using the Qubit Fluorometer (Invitrogen), and RNA integrity was assessed with an Agilent Bioanalyzer 2100 (Agilent Technologies, Santa Clara, CA, USA) utilizing the Agilent RNA 6000 Nano Kit to ensure the quality of the extracted RNA. For amplification, 500 ng of total RNA was utilized in the Ovation FFPE WTA System (NuGEN, San Carlos, CA, USA). Fragmentation and labeling of the amplified RNA were carried out using the NEBNext® Ultra™ II DNA Library Prep Kit (Illumina). The quality and quantity of the resulting libraries were assessed using Qubit (Invitrogen, Carlsbad, CA, USA) and the Agilent Bioanalyzer 2100. Subsequently, all libraries were sequenced on a DNBSEQ‐T7RS (MGI) platform with 100‐bp paired‐end reads. Base call files generated during sequencing were converted using cal2Fastq to the fastq format. Raw data were normalized and further procession using fastp (version 0.20.1).

### 4.3 Tissue preparation and WSIs patching

A total of 2557 H&E‐stained histopathology slides from 925 patients were accessed through TCGA's GDC, and 1557 slides from 95 patients were retrospectively acquired from SYSMH. These FFPE breast tumor slides, obtained 1–4 weeks before chemotherapy or targeted therapy, were digitized. WSIs, with ∼10 gigapixels each, were preprocessed for analysis.

The biopsy tissue in each WSI was segmented using the CLAM WSI analysis toolbox.^45^ The processing pipeline for digitized slides involved automated tissue segmentation. WSIs were downsampled (e.g., 32‐fold) and converted from RGB to HSV. A binary mask for tissue regions was created using thresholding on the saturation channel, followed by median blurring and morphological closing. Detected foreground contours were filtered by area threshold. Segmentation masks were available for visual inspection, and a text file with key parameters was generated, allowing for manual adjustments if needed. Postsegmentation, the algorithm extracted 256 × 256 patches from the segmented foreground at user‐specified magnification. These patches, along with coordinates and slide metadata, were saved in the hdf5 format. The number of patches varied from hundreds for biopsy slides at ×20 magnification to hundreds of thousands for large resection slides at ×40 magnification.

### 4.4 Transcriptome sequencing data analysis

Differential expression analysis of microarray data was performed using the limma R package,^46^ and differential expression analysis of RNA‐sequencing data was based on popular R packages (www.r‐project.org) for analysis to evaluate robustness, namely, the DESeq2^47^ and edgeR.^48^

Unsupervised clustering analysis was used to identify different lncRNA patterns and immune patterns. The patients were divided into different subtypes using the R package “ConsensusClusterPlus” for further analysis.^49^ A total of 1000 iterations were conducted and a resample rate of 80% was defined to ensure the stability of classification, and the cumulative distribution function curve was used to determine the clustering number.

To identify the enriched terms in Gene Ontology and Kyoto Encyclopedia of Genes and Genomes, Gene Set Enrichment Analysis (GSEA) was conducted via the R package “clusterProfiler^50^”. GSEA is a statistical method used to demonstrate significant differences among subtypes and the signaling pathways regulated by lncRNA and immune cells using TCGA data.

The infiltration level of the different immune‐cell populations was determined by ssGSEA in the R Bioconductor package Gene Set Variation Analysis using default parameters according to the original study and a previous study.^51^, ^52^ The ssGSEA algorithm is a rank‐based method that defines a score representing the degree of absolute enrichment of a particular gene set in each sample. The ssGSEA scores for most immune‐cell populations were obtained using the gene sets. We used hierarchical clustering to identify immune subtypes of breast cancer based on the ssGSEA scores of 28 immune‐cell types, including CD56‐bright NK cells, effector memory CD4^+^ T cells, eosinophils, CD56‐dim NK cells, type 17 T helper cells, activated B cells, monocytes, memory B cells, activated CD4^+^ T cells, type 2 T helper cells, plasmacytoid dendritic cells, neutrophils, macrophages, effector memory CD8^+^ T cells, MDSCs, immature B cells, T follicular helper cells, immature dendritic cells, mast cells, type 1 T helper cells, activated dendritic cells, central memory CD4^+^ T cells, gamma delta T cells, central memory CD8^+^ T cells, regulatory T cells, activated CD8^+^ T cells, and NK T cells.

The exploring mutation signatures of the subtypes, MAF data were obtained from GDC portal and analyzed via the R package “maftools” according to the guideline from Bioconductor. Oncoplots were performed for summarizing mutation landscape. And function “oncodrive” which is based on algorithm oncodriveCLUST,^53^ was used to identify driver mutation. Clinical enrichment analysis was performed also using “clinicalEnrichment” of this package.

### 4.5 Developing a pathology‐based AI model for lncRNA–metabolism subtype prediction

This study presented a sophisticated model tailored for lncRNA–metabolism class prediction, leveraging a novel bag‐of‐patches approach. The methodology encompassed the utilization of digitized high‐resolution histology slides, organized into bags of patches, as input data for a pathology‐based AI model. To enhance the model's performance, a two‐step process was adopted: (1) initially, transfer learning was applied with a feature extraction stage, utilizing frozen parameters to ensure stability and efficiency; (2) next, the model underwent fine‐tuning with a modified structure, allowing trainable parameters to adapt and optimize performance.

To perform feature extraction, we used a customized CNN based on pretrained ResNet50 architecture trained on ImageNet dataset.^54^, ^55^ The subsequent modified module comprised an embedding section with a fully connected layer, seamlessly integrating an attention‐gated mechanism. The attention‐gated module was designed with two linear projections, each utilizing distinct activation functions—rectified linear unit and sigmoid—as the query and key, respectively.^56^ Subsequently, the softmax operation was applied to compute the product of the two outcomes, resulting in attention scores. These scores were then used as multipliers against the attention scores assigned to the entire set of patches.

The resulting high‐dimensional features were then passed to the final classifier layer that consisted of a linear layer. This layer processed the aggregated features to produce probabilities for different classifications, providing a robust prediction framework for lncRNA–metabolism subtypes.

### 4.6 Details of developing pathology‐based AI model for lncRNA–metabolism subtype prediction

#### 4.6.1 Feature extraction

A pretrained ResNet50 model was used for feature extraction from image patches. Adaptive mean‐spatial pooling after the third residual block converted 256 × 256 patches into 1024‐dimensional vectors. This process, using a batch size of 128 across multiple GPUs, reduced training time and costs, allowing for rapid model training on thousands of WSIs. Low‐dimensional features enabled processing all patches in a slide on a single GPU, avoiding patch sampling and noisy labels.

#### 4.6.2 Gated attention mechanism

We proposed the use of a weighted average of patches (low‐dimensional embeddings), where weights were determined by a neural network. To ensure that the sum of weights was 1 and that they were invariant to the size of a bag, we applied the softmax nonlinearity. Additionally, we used the hyperbolic tangent (tan*h*(·)) element‐wise nonlinearity to include both negative and positive values for proper gradient flow. We also employed the gating mechanism^57^ in conjunction with tan*h*(·) nonlinearity as follows:

$$a=\mathrm{softmax}(W^{T}\cdot (\tan h\left(U\cdot h^{T}\right)⊙\left(\mathrm{sigm}\left(V\cdot h^{T}\right)\right),$$

where h is the input feature, $W\in R^{L};U,V\in R^{L\times M}$ are parameters, ⊙ denotes element‐wise multiplication, and sigm(·) is the sigmoid element‐wise nonlinearity. The gating mechanism introduces a learnable nonlinearity that potentially removes the troublesome linearity in tan*h*(·).

### 4.7 Developing a multimodal AI model for survival risk prediction

To predict prognostics, we engineered a cutting‐edge multimodal AI model that encompassed the same CNN architecture, an attention‐module, concatenation fusion, and a conclusive regressor. This model harmoniously amalgamated intricately tailored nested neural networks designed for pathology images, lncRNA data, immune‐cell ssGSEA scores, and clinical information.

To ensure meticulous feature embedding, dedicated encoders played a pivotal role in our model architecture. Initial features extracted from WSIs stemmed from the modified CNN model, capturing inherent intricacies. In optimizing the attention‐module's efficiency, we innovatively devised a dynamic query‐router (QR). This QR incorporated two multilayer perceptron (MLP) layers, each complemented by exponential linear unit activation functions^58^ and a Gumbel‐Softmax layer for the purpose of sampling binary routes.

The input query was first transformed by an MLP layer, then dynamically integrated into postprocessing via another MLP layer, enhancing adaptability. Gumbel‐Softmax sampling on the modified query generated binary route probabilities, which masked the input query to produce the final masked version. This process enabled dynamic information routing and adaptive feature selection. Features were then processed through an attention module with the modified query to obtain latent features based on attention scores.

For comprehensive integration and optimal data retention, we used concatenation to merge modality features into a unified representation. This was followed by a fully connected layer that served as a regressor, producing three key values: the status probability (0 or 1) and the time‐based risk level score. To refine risk assessment, we calculated a robust risk score by multiplying the maximum status probability with the time‐based risk level score, thereby improving model accuracy and reliability.

### 4.8 Details of developing multimodal AI model for survival risk prediction

#### 4.8.1 Query‐router

The query attention employed a sequence‐adaptive pathway router to query pathway routes for attention. The router generated a binary route $R\in {\left\{0,1\right\}}^{L}$ to determine whether a bag token would be part of the query pathway or not. All elements in the route were initialized to 1 and were progressively updated during training. To mitigate potential disturbance caused by local drifted interest, it was crucial to incorporate global information in the route generation. Average pooling was applied to all of the preserved query tokens routed by R, and the global sequential representation was produced using an MLP module. Subsequently, this global representation was combined with the inputs, employing a residual connection to maintain the original input information. Finally, they were fed to another MLP layer to predict the probabilities of keeping or dropping the query tokens. The procedure can be formulated as follows:

$$Q_{\mathrm{emb}}^{\mathrm{out}}=Q^{\mathrm{in}}+Q^{\mathrm{in}}⊙\mathrm{MLP}\left(Q^{\mathrm{in}}\right)$$

$$r=\mathrm{softmax}\left(\mathrm{MLP}\left(Q_{\mathrm{emb}}^{\mathrm{out}}\right)\right)\in_{R}L\times 2,$$

where Q is the query feature, ʘ is the Hadamard product, and r=[α,1−α], where the logit α denotes the probability that the query token is kept for the input pathway.

#### 4.8.2 Gumbel‐Softmax sampling from *π* for router

The goal was to generate the binary route from r. However, sampling directly from r was nondifferentiable and impeded gradient‐based training. Thus, we applied the Gumbel‐Softmax technique to such sampling.^59^ Gumbel‐Softmax is an effective method to approximate the original nondifferentiable sample from a discrete distribution with a differentiable sample from a Gumbel‐Softmax distribution. With this design, if a query token failed to be routed in a certain block, it permanently lost the privilege to be part of the attention pathway in the subsequent feed‐forward procedure, constituting a hierarchical pathway router strategy. Figure 1A illustrates the detailed graphical summary of the experimental design.

### 4.9 Statistical analysis

Survival curve analysis was based on Kaplan–Meier plots. The results are displayed as HRs and *p* values from a log‐rank test. Kaplan–Meier curves with log‐rank tests were used to determine survival differences. Significance thresholds were set at two‐sided *p* values below 0.05 for all conducted analyses. Patient stratification into high‐ and low‐risk groups was achieved using optimal cutoff values identified by the R package survminer. The prognostic or predictive accuracy of the generated signatures was assessed through ROC curve analysis. Sensitivity and specificity were evaluated using the area under the ROC curve (AUC), providing an effective measure to assess the predictive performance of the signatures. These statistical computations were performed using R software (version 4.3.1). WSI processing and feature extraction were performed using Python (version 3.7.7). For WSI processing and segmentation, OpenSlide‐Python (1.2.0), OpenSlide (3.0), and OpenCV‐Python (version 4.1.1) were employed. PyTorch (1.7.1+cu101) was utilized to train deep learning models on GPUs.

## AUTHOR CONTRIBUTIONS

All authors had full access to all the data in the study and take responsibility for the integrity of the data and the accuracy of the data analysis. YF. Y., GY. C., WH. O., and ZH. Q. did the acquisition, analysis, and interpretation of data and provided administrative, technical, and material support. K. Z. and HR. Y. provided study supervision. All authors drafted and revised the manuscript. All authors have read and approved the final manuscript.

## CONFLICT OF INTEREST STATEMENT

Author Kang Zhang is an Editorial board member of MedComm. Author Kang Zhang was not involved in the journal's review of or decisions related to this manuscript. The other authors declared no conflict of interest.

## ETHICS STATEMENT

The study was conducted in accordance with the Declaration of Helsinki and approved by the Ethics Committee of Sun Yat‐sen Memorial Hospital, Sun Yat‐sen University (SYSKY‐2024‐363‐01). The informed patient consent was waived as this study is a retrospective study.

## Supporting information



Supporting Information

## Data Availability

The RNA‐seq data are available in GEO with accession number GSE189371. The original code is available in GitHub at https://github.com/DeepClinMed/IM_PGM/tree/main. Any additional information required in this paper is available from the lead contact upon request.
