# Supplementary material for: Multimodal data fusion AI model uncovers tumor microenvironment immunotyping heterogeneity and enhanced risk stratification of breast cancer
Source: MedComm (2020). 2024 Dec 11;5(12):e70023. doi: 10.1002/mco2.70023 (PMC11635117; doi:10.1002/mco2.70023)
Supplement: Supplementary file 1 — Supporting Information [file MCO2-5-e70023-s001.docx]

**Multimodal Data Fusion AI Model Uncovers Tumor Microenvironment Immunotyping Heterogeneity and Enhanced Risk Stratification of Breast Cancer**

Yunfang Yu^1,2^, Gengyi Cai^1^, Ruichong Lin^3^, Zehua Wang^3^, Yongjian Chen^4^, Yujie Tan^1^, Zifan He^1^, Zhuo Sun^5^, Wenhao Ouyang^1*^, Herui Yao^1*^, Kang Zhang^2,5,6,7*^

**Author Affiliation**

^1^Guangdong Provincial Key Laboratory of Malignant Tumor Epigenetics and Gene Regulation, Department of Medical Oncology, Breast Tumor Centre, Phase I Clinical Trial Centre, Sun Yat-sen Memorial Hospital, Sun Yat-sen University, Guangzhou, China.

^2^Faculty of Medicine, Macau University of Science and Technology, Taipa, Macao, PR China.

^3^Faculty of Innovation Engineering, Macau University of Science and Technology, Macau, China.

^4^Dermatology and Venereology Division, Department of Medicine Solna, Center for Molecular Medicine, Karolinska Institute, Stockholm, Sweden.

^5^Institute for Advanced Study on Eye Health and Diseases, Wenzhou Medical University, Wenzhou, China.

^6^Guangzhou National Laboratory, Guangzhou, China.

^7^Zhuhai International Eve Center, Zhuhai People's Hospital and the First Affiliated Hospital of Faculty of Medicine, Macau University of Science and Technology and University Hospital, Zhuhai, China.

**Data Supplement Content**

**Figure S1.** Identification of lncRNA and immune cells features

**Figure S2.** Forest Plot shows four overall survival related immune cells and ten overall survivals related lncRNAs.

**Figure S3**. Oncoplot shows mutations genes landscape of four subtypes.

**Figure S4.** Bar plot shows groupwise comparisions of identification of enriched mutations for four classes.

**Figure S5.** Dot plot shows cancer driver genes identification based on positional clustering.

**Figure S6.** Box plot shows immune cell infiltration level of each subtype**.**

**Figure S7.** Heatmap shows different signatures of each subtype.

**Figure S8.** ROC curves of single-omics, pathology slides based-model for prediction of prognostics.

**Figure S9.** Features weights in multimodal data based-model training.

**Table S1.** Clinicopathologic characteristics in the testing cohort.

**Figure S1.** **Identification of features related to lncRNAs and immune cells.**

**
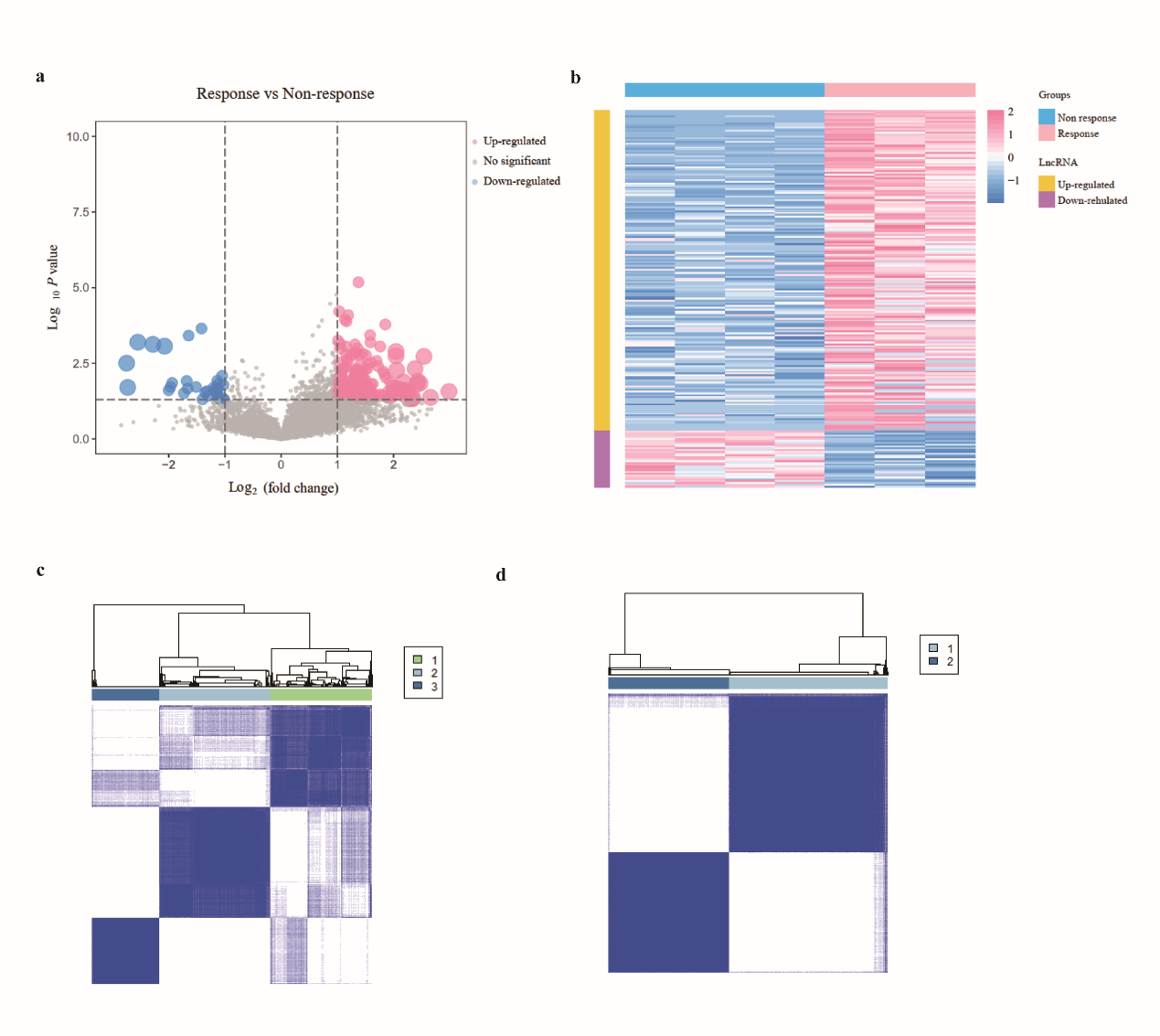
** (A) Volcano plot shows the differentially expressed lncRNAs (log2 fold-change ≥ 1) in immunotherapy responders and non-responders. (B) Heat map shows the differentially expressed lncRNAs in immunotherapy responders and non-responders. (C) Non-negative matrix factorization clustering algorithm by 925 breast cancer samples with sequenced lncRNAs. (D) Non-negative matrix factorization clustering algorithm by 28 types of immune cells.

**Figure S2. Forest Plot shows four overall survival related immune cells and ten overall survivals related lncRNAs.**

**
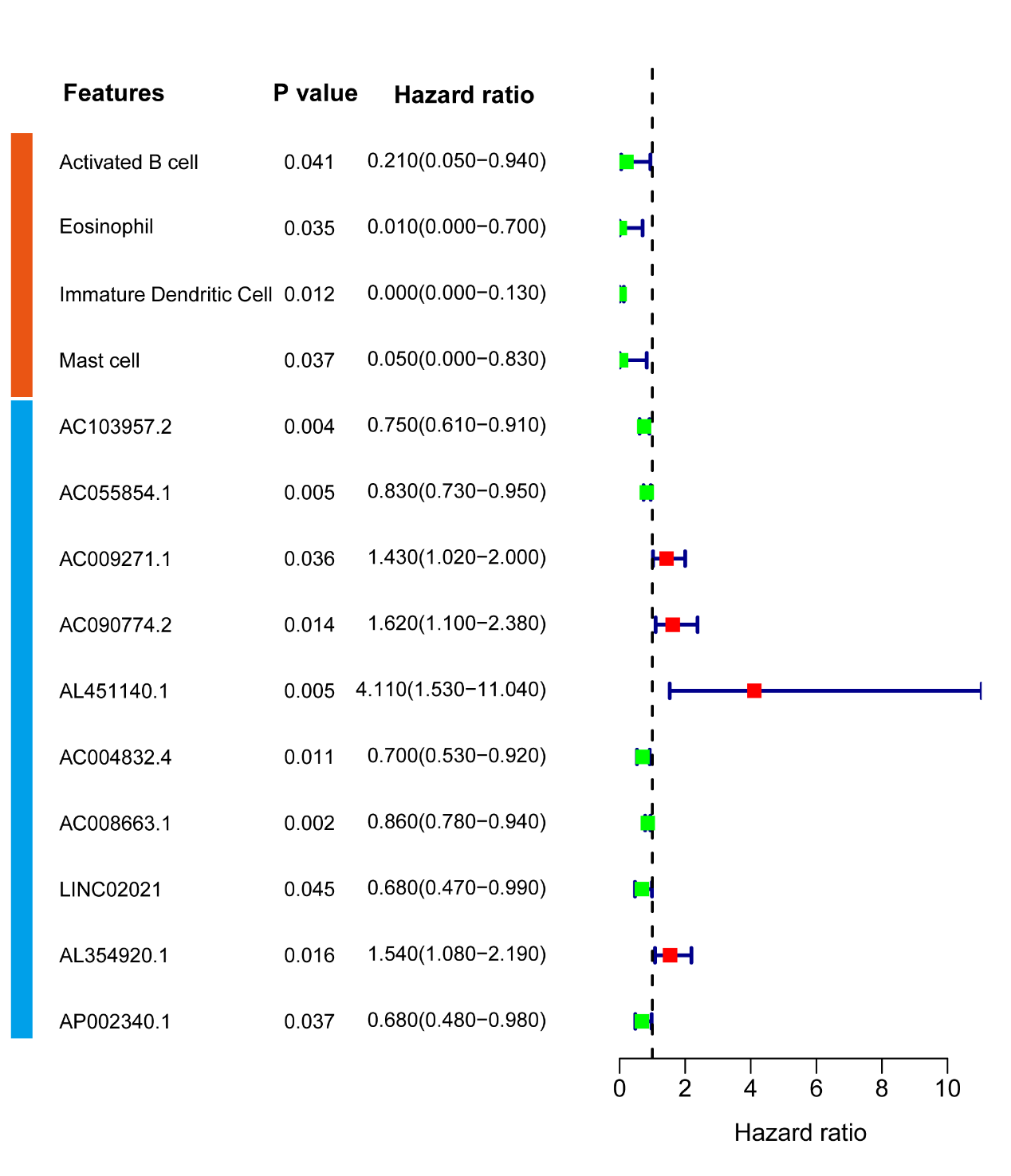
**

**Figure S3. Oncoplot shows mutations genes landscape of four subtypes.**

**
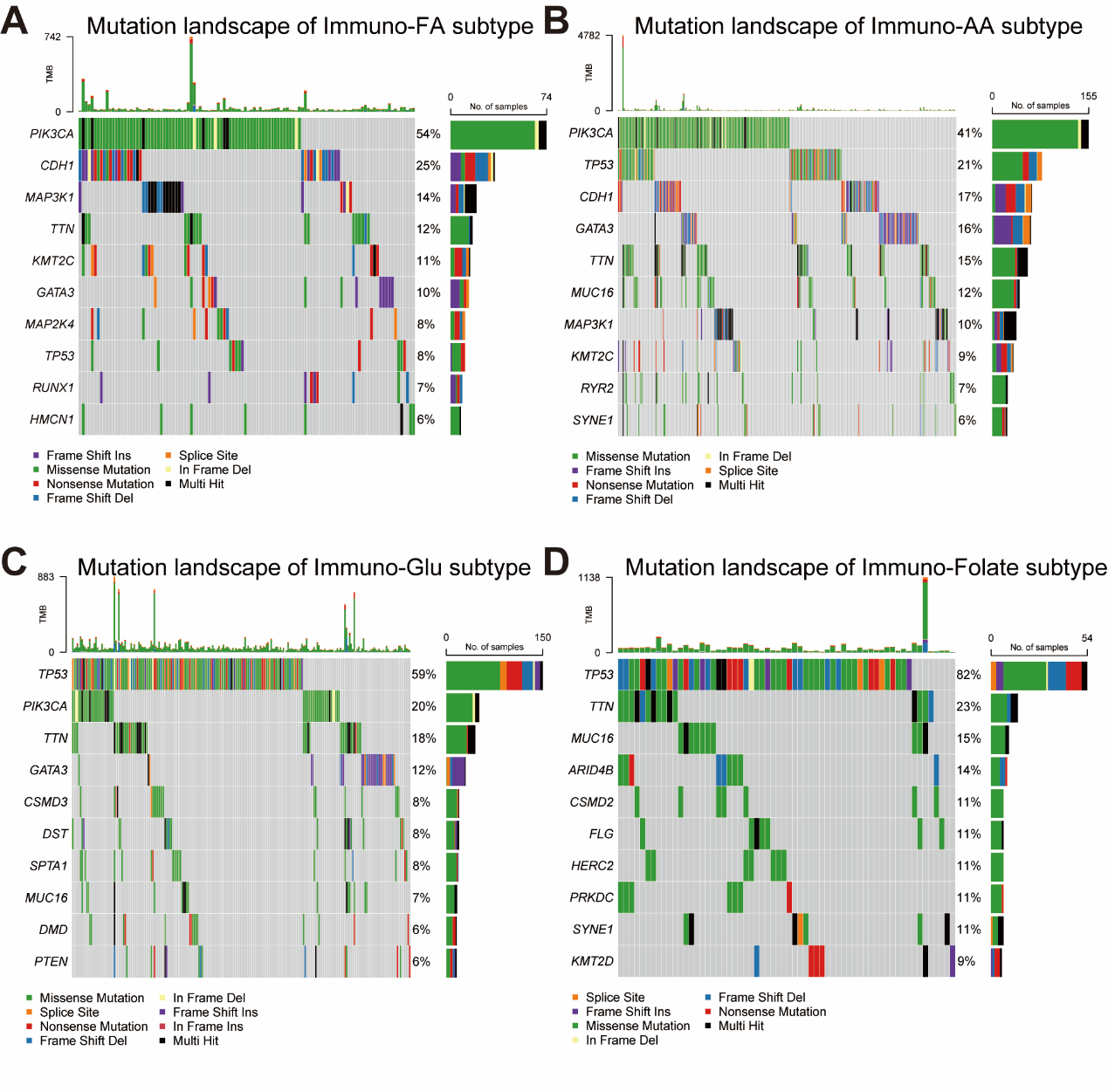
**

**Figure S4. Bar plot shows groupwise comparisions of identification of enriched mutations for four classes.**

**
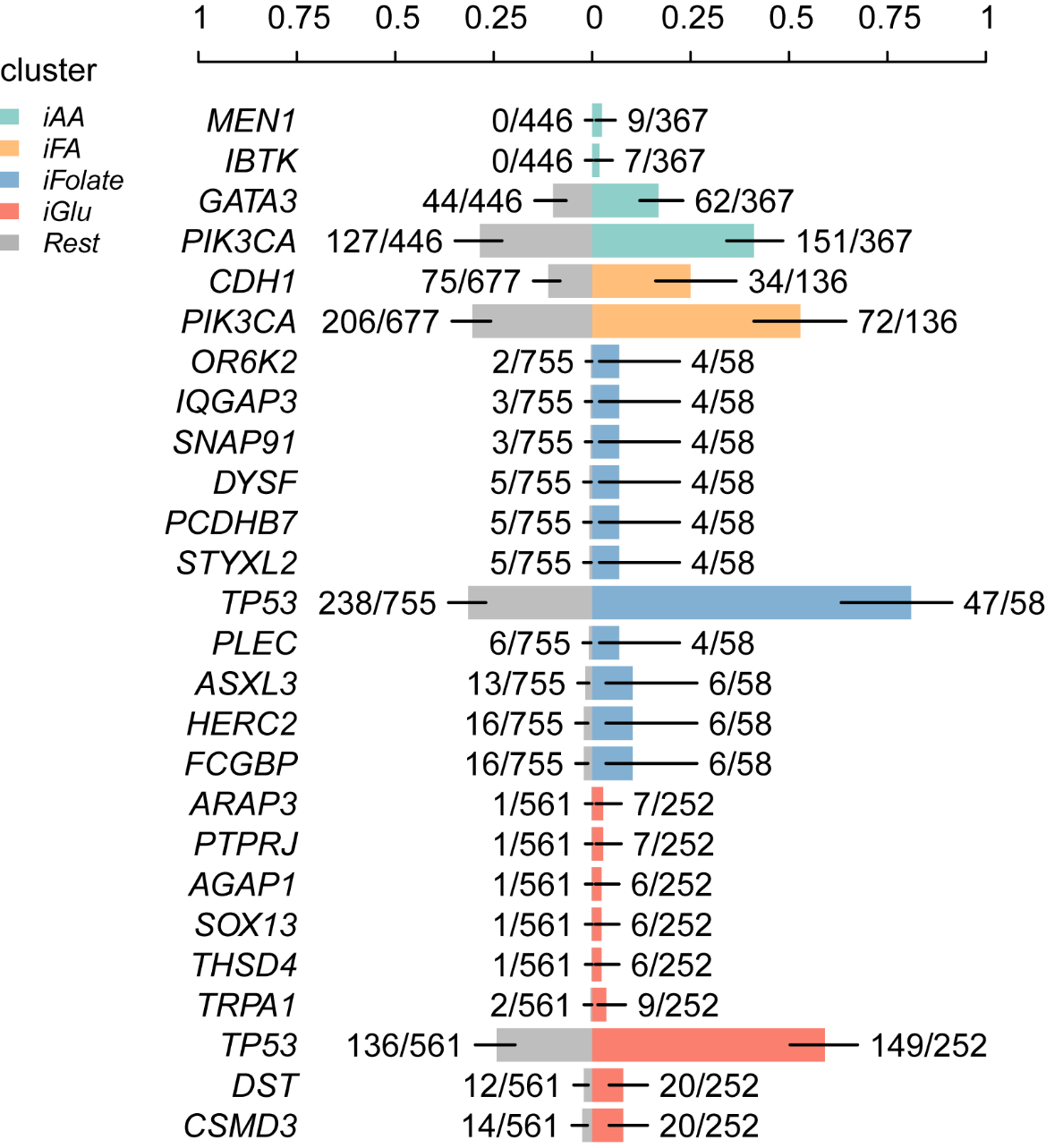
**

**Figure S5. Dot plot shows cancer driver genes identification based on positional clustering.**


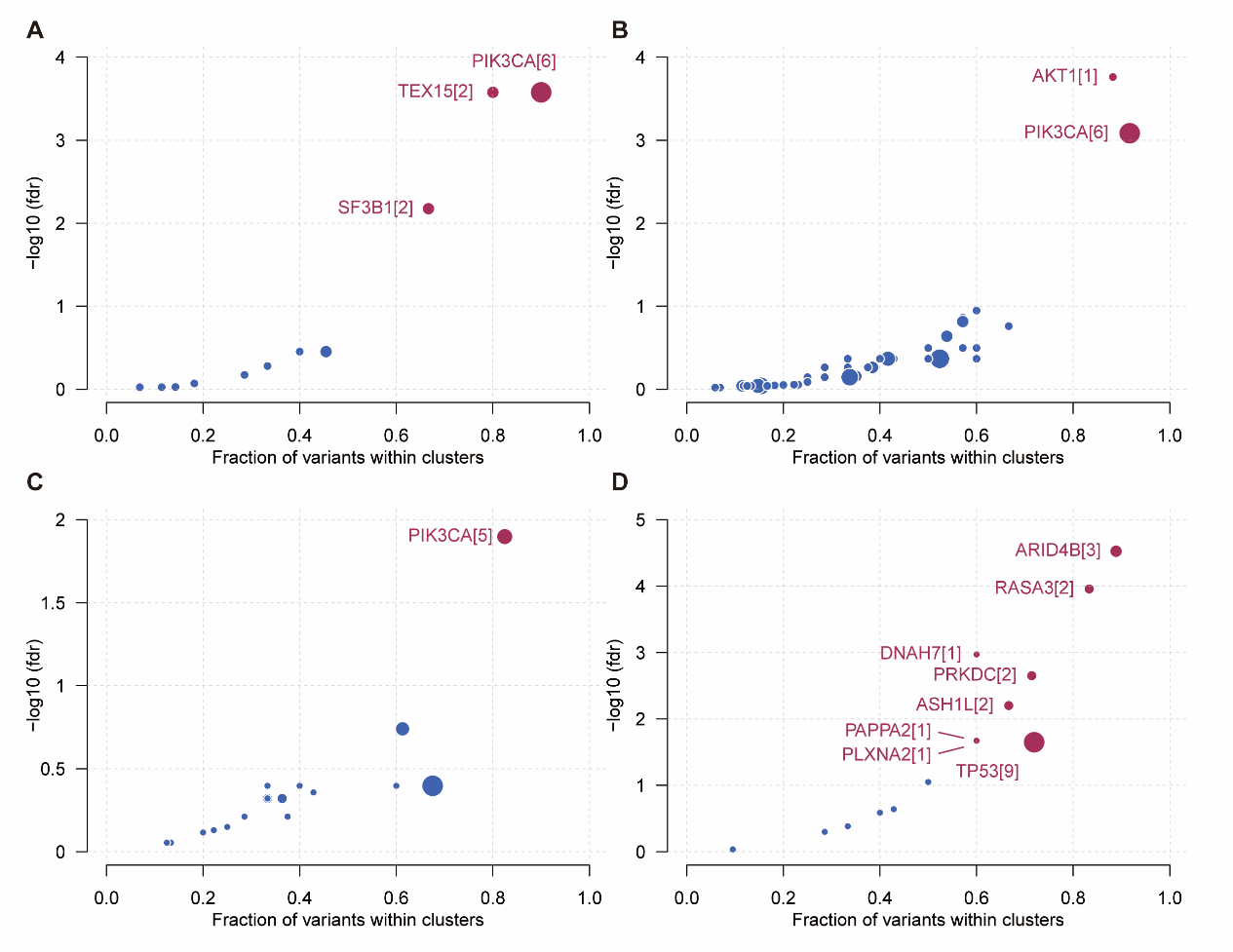


Cancer driver genes identification of (A) Immuno–FA, (B) Immuno–AA, (C) Immuno–Glu, (D) Immuno–Folate, based on positional clustering. Red dot represented potential driver genes of the subtypes.

**Figure S6. Box plot shows immune cell infiltration level of each subtype.**


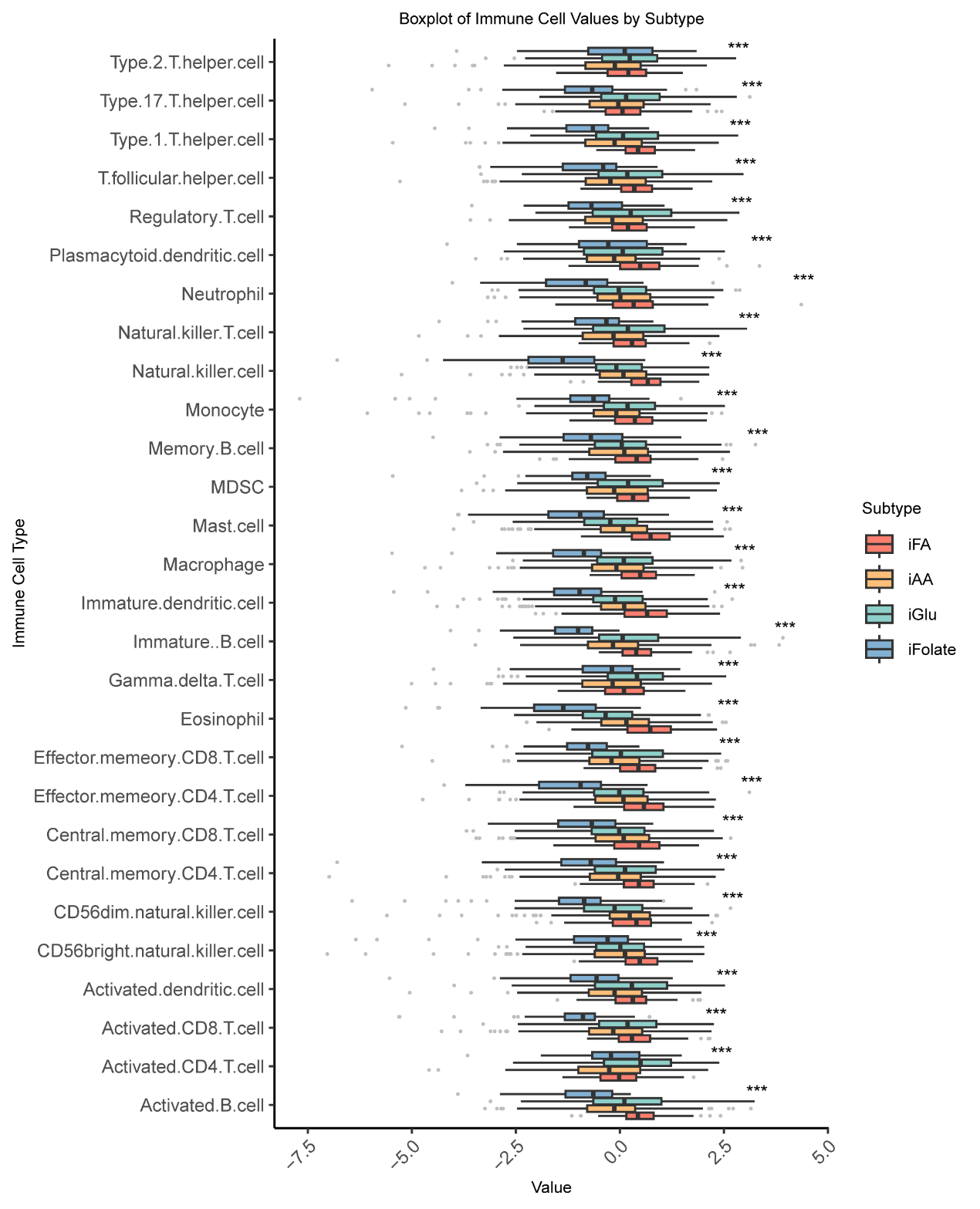


**Figure S7. Heatmap shows different signatures of each subtype.**


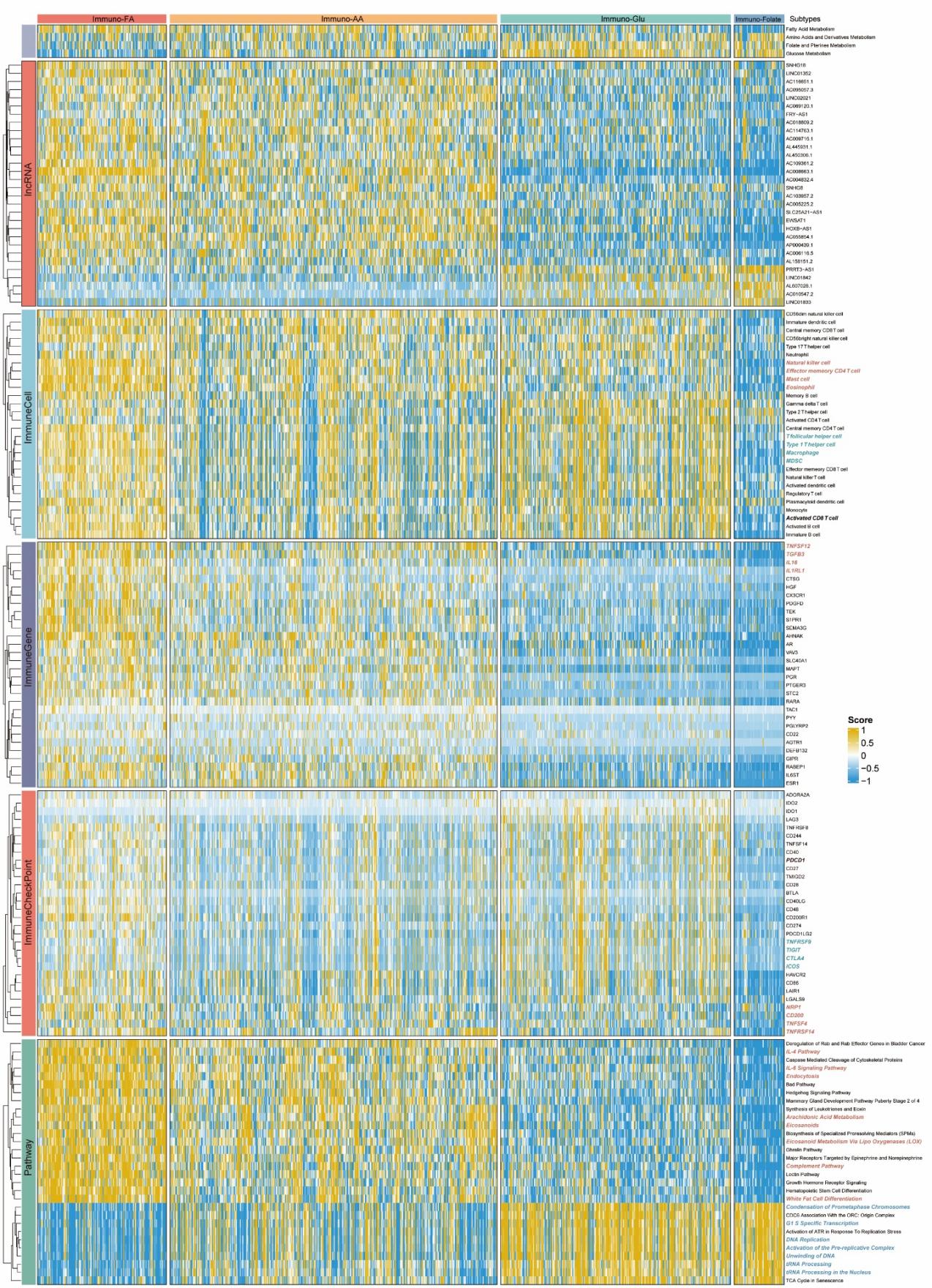
Heatmap of the major metabolism score, the immunotherapy-associated lncRNA expression, immune cells, immune checkpoints gene expression, immune gene expression, enriched pathways in GSEA analysis for each lncRNA-metabolism class. Each cell represents the expression or scores of each patient in the item.

**Figure S8.** ROC curves of single-omics, pathology slides based-model for prediction of prognostics.


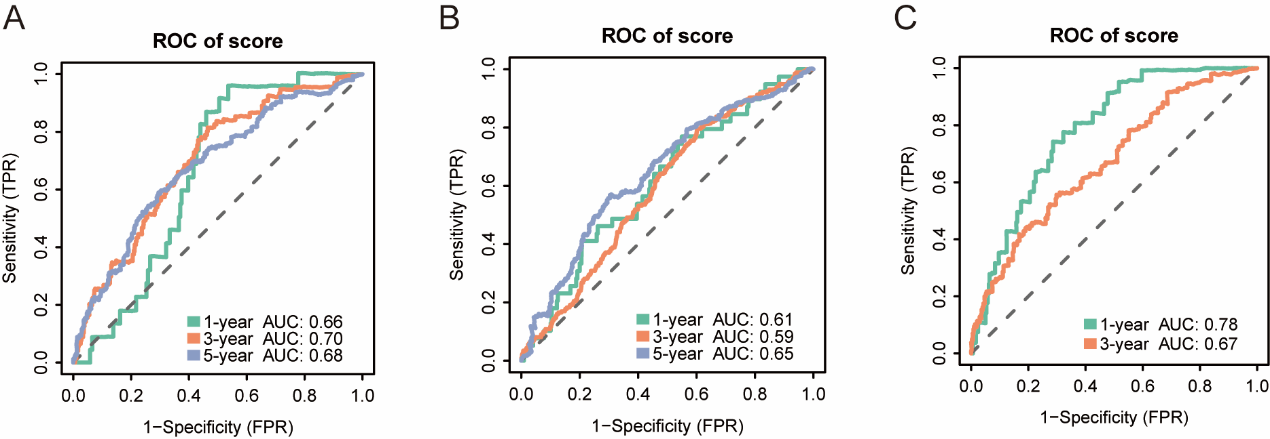


ROC curves for survival prediction by single-omics, pathology slides based-model in the training cohort (A), internal validation cohort (B), and independent testing cohort (C). AUC = Area under the curve; TPR = True-positive rate; FPR = False-positive rate.

**Figure S9.** Features weights in multimodal data based-model training.


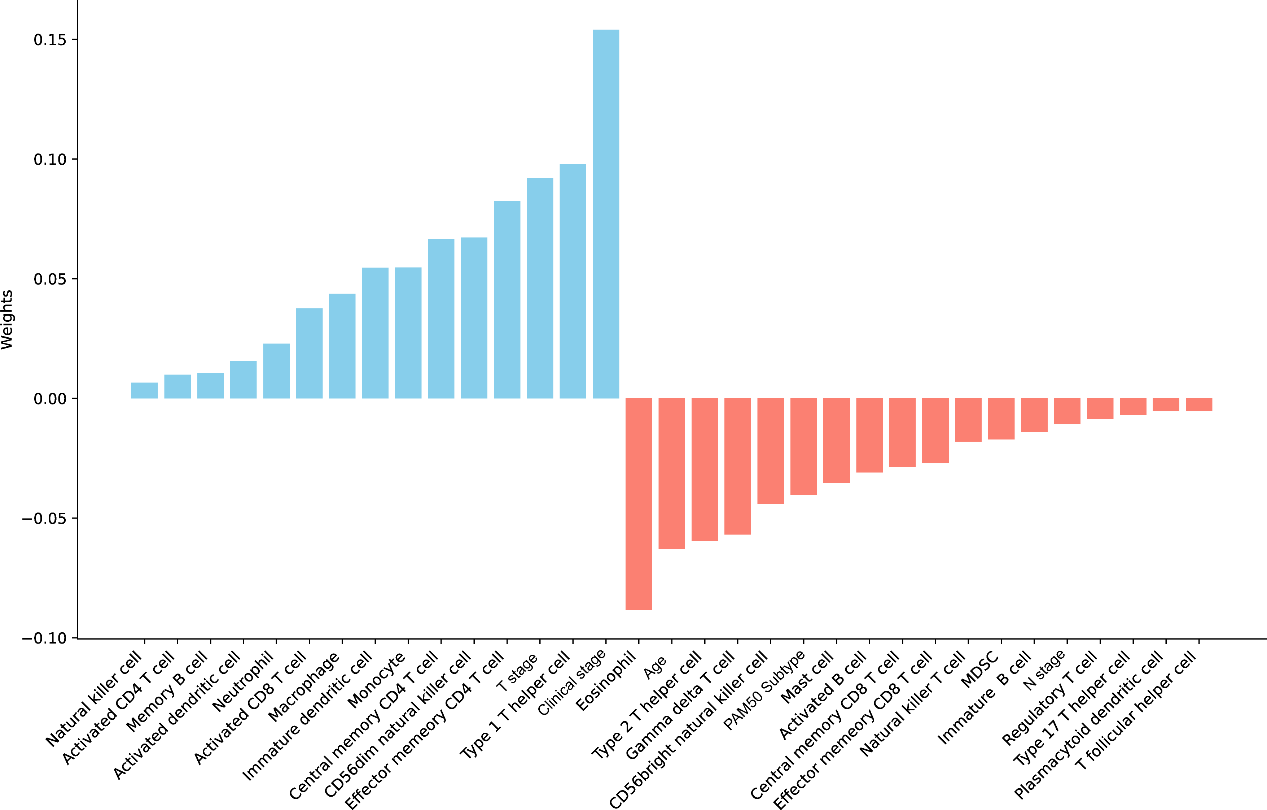


Bar plots illustrate the weights assigned to clinical records and different cell types during the training of a multi-omics model. The vertical axis represents the weight values, which indicate the importance of each feature in the model. Positive weights suggest a higher significance in the model's predictive power, whereas negative weights indicate a potentially inverse relationship.

**Table S1. Clinicopathologic characteristics in the testing cohort.**

| **Baseline** | **All patients [No. (%)]** |
| --- | --- |
| **Total** | 95 (100.00%) |
| **Age (Median ± SD), Years** | 50.0 ± 9.8 |
| **Number of Tumors** |  |
| 1 | 77 (81.05%) |
| 2 | 18 (18.95%) |
| **Clinical T Stage** |  |
| T1 | 31 (32.63%) |
| T2 | 55 (57.89%) |
| T3 | 7 (7.37%) |
| T4 | 2 (2.11%) |
| **Clinical N Stage** |  |
| N0 | 51 (53.68%) |
| N1 | 43 (45.26%) |
| N2 | 1 (1.05%) |
| **Clinical TNM** |  |
| I | 22 (23.16%) |
| II | 65 (68.42%) |
| III | 8 (8.42%) |
| **Pathological GRADE** |  |
| 1 | 5 (5.26%) |
| 2 | 41 (43.16%) |
| 3 | 49 (51.58%) |
| **Pathological T Stage** |  |
| 1 | 52 (54.74%) |
| 2 | 40 (42.11%) |
| 3 | 3 (3.16%) |
| **Pathological N Stage** |  |
| 0 | 48 (50.53%) |
| 1 | 23 (24.21%) |
| 2 | 13 (13.68%) |
| 3 | 11 (11.58%) |
| **Pathological TNM** |  |
| I | 32 (33.68%) |
| II | 38 (40.00%) |
| III | 25 (26.32%) |
| **Molecular Subtype** |  |
| Luminal A | 11 (11.58%) |
| Luminal B | 63 (66.32%) |
| Her-2 Positive | 18 (18.95%) |
| Triple Negative | 3 (3.16%) |
| **Follow-up Time(Median ± SD), Months** | 36.63±7.46 |
